# Sequence Skill Acquisition and Off-Line Learning in Normal Aging

**DOI:** 10.1371/journal.pone.0006683

**Published:** 2009-08-19

**Authors:** Rachel M. Brown, Edwin M. Robertson, Daniel Z. Press

**Affiliations:** Department of Neurology, Beth Israel Deaconess Medical Center, Boston, Massachusetts, United States of America; Claremont Graduate University, United States of America

## Abstract

It is well known that certain cognitive abilities decline with age. The ability to form certain new declarative memories, particularly memories for facts and events, has been widely shown to decline with advancing age. In contrast, the effects of aging on the ability to form new procedural memories such as skills are less well known, though it appears that older adults are able to acquire some new procedural skills over practice. The current study examines the effects of normal aging on procedural memory more closely by comparing the effects of aging on the encoding or acquisition stage of procedural learning versus its effects on the consolidation, or between-session stage of procedural learning. Twelve older and 14 young participants completed a sequence-learning task (the Serial Reaction Time Task) over a practice session and at a re-test session 24 hours later. Older participants actually demonstrated more sequence skill during acquisition than the young. However, older participants failed to show skill improvement at re-test as the young participants did. Age thus appears to have a differential effect upon procedural learning stages such that older adults' skill acquisition remains relatively intact, in some cases even superior, compared to that of young adults, while their skill consolidation may be poorer than that of young adults. Although the effect of normal aging on procedural consolidation remains unclear, aging may actually enhance skill acquisition on some procedural tasks.

## Introduction

Normal aging leads to declines in certain cognitive abilities while leaving other abilities intact. It is known that aging particularly impairs the formation of certain types of declarative memories, for instance, recall and recognition of new facts and events [1], [2]. In contrast, the effect of aging on the ability to form new procedural memories such as motor skills has received less attention in the aging literature. Existing studies show that aging is accompanied by general declines in motor execution such as reaction time speed and accuracy [3]. However, older adults retain the ability to improve on certain motor tasks over an initial period of practice, or during encoding, the first stage of procedural memory. For instance, in a task of fine motor movement and manipulation of objects, older subjects improved their motor execution speed over practice [4]. Older adults have also shown comparable performance improvements to young adults during encoding of a motor sequence. Participants completed a version of the serial reaction time task (SRTT) in which they learned a sequence of finger movements using visual cues, and their performance was measured by response time. After performing the sequence over a series of practice blocks, older and young participants demonstrated comparable practice effects as indicated by speeded reaction times. In addition, both age groups demonstrated comparable sequence-specific learning as indicated by an increase in response times when switching from sequential to random finger movements [5], [6].

Older participants thus appear to be able to learn certain procedural tasks as effectively as young adults during the encoding, or acquisition, phase of procedural learning since they show similar improvements during initial training. However, further skill can potentially be obtained during the consolidation phase of procedural memory, or the stage following acquisition. Recent studies have shown that college-age subjects can continue to increase their level of skill on sequence tasks between practice sessions [7], [8]. This between-session improvement, termed “off-line” learning, is one behavioral expression of procedural consolidation. Young adults continue to acquire skill on a sequence-learning task over a period of 12 waking hours without practice on the task [9]. We sought to examine the comparative effects of aging on procedural acquisition and on procedural consolidation as indicated by off-line learning on a task of procedural learning.

We tested a group of older and younger adults on the Serial Reaction Time Task (SRTT) on two testing sessions separated by 24 hours, including both wake and sleep. This task requires participants to respond via button-pressing to a series of dots that appear in one of four spatial locations on a computer screen. These spatial cues appear in blocks of trials with either a random or a sequential order. By comparing participants' reaction time on sequential versus random trials, sequence-specific learning can be assessed both within sessions (acquisition) and between sessions (off-line learning). We sought to compare the affects of normal aging on both acquisition and off-line learning of this procedural task.

## Methods

### Participants

Thirty-two healthy adults were recruited for this study. They included 10 female and 8 male young adults (*n* = 18) and 9 female and 5 male older adults (*n* = 14). Fourteen young adults (*M* = 20.4 years of age, *SD* = 1.6) and 12 older adults (*M* = 58.3 years of age, *SD* = 3.8) were included for analyses (*N* = 26). Four young and two older participants were excluded because they either generated unusable data (*n* = 3 young), showed outlying scores of more than three standard deviations away from the mean on the primary behavioral task (*n* = 1 young, *n* = 1 older), or did not perform the task properly (*n* = 1 older). All participants were right-handed according to their reports on the Edinburgh Handedness Questionnaire, and all participants reported being free of neurological and psychiatric illnesses. Young participants scored marginally but significantly better on the Mattis dementia rating scale (*M* = 143.82/144, *SD* = 0.6) than older participants (*M* = 142.33/144, *SD* = 1.8), *t*_(21)_ = −2.57, *p*<0.05), although all participants scored within the normal range. (Three young participants did not complete the Mattis scale). Older participants completed significantly more years of education (*M* = 19.3, *SD* = 3.9) than young participants (*M* = 12.3, *SD* = 1.1, *t*_(24)_ = 6.44, *p*<.0001), likely due to the fact that most of the young had not yet completed their college education. Older participants were recruited from the greater Boston area via fliers that were posted around the testing site as well as via online postings. Younger adults were recruited from local colleges (primarily Boston University). All subjects received $30 in cash as compensation. All subjects underwent both written and verbal informed consent. The study was approved by the Committee on Clinical Investigations of Beth Israel Deaconess Medical Center, Boston, MA.

### Procedure

All subjects performed the Serial Reaction Time Task (SRTT), a procedural sequence-learning measure [10]. Subjects sat in front of a computer screen with their right hand resting on a button box with four buttons in a horizontal array. Participants then saw blue dots appear one at a time in one of four horizontal positions across a white computer screen. Subjects were required to press the button that corresponded to the position of the dots as quickly and accurately as they could. Each dot presented was set to disappear as soon as participants pressed the correct corresponding button, and the interval between each correct response and the next stimulus was set to 400 milliseconds.

We used an SRTT task design that was similar to that used by Curran [11] in which random and sequence trials were present in each block. This allowed sequence-specific learning to be measured over each individual block of practice. Random trial orders were pre-determined by the investigators such that there were no repetitions (i.e. 1-4-2-2) and no triplets shared by sequential trials. Random trials were therefore pseudorandom (though we will use the term “random” throughout the rest of the paper). Sequential trials followed a 12-item sequential order (2-3-1-4-3-2-4-1-3-4-2-1, 1 corresponding to the left-most position, and 4 corresponding to the right-most position).

**Figure 1:**
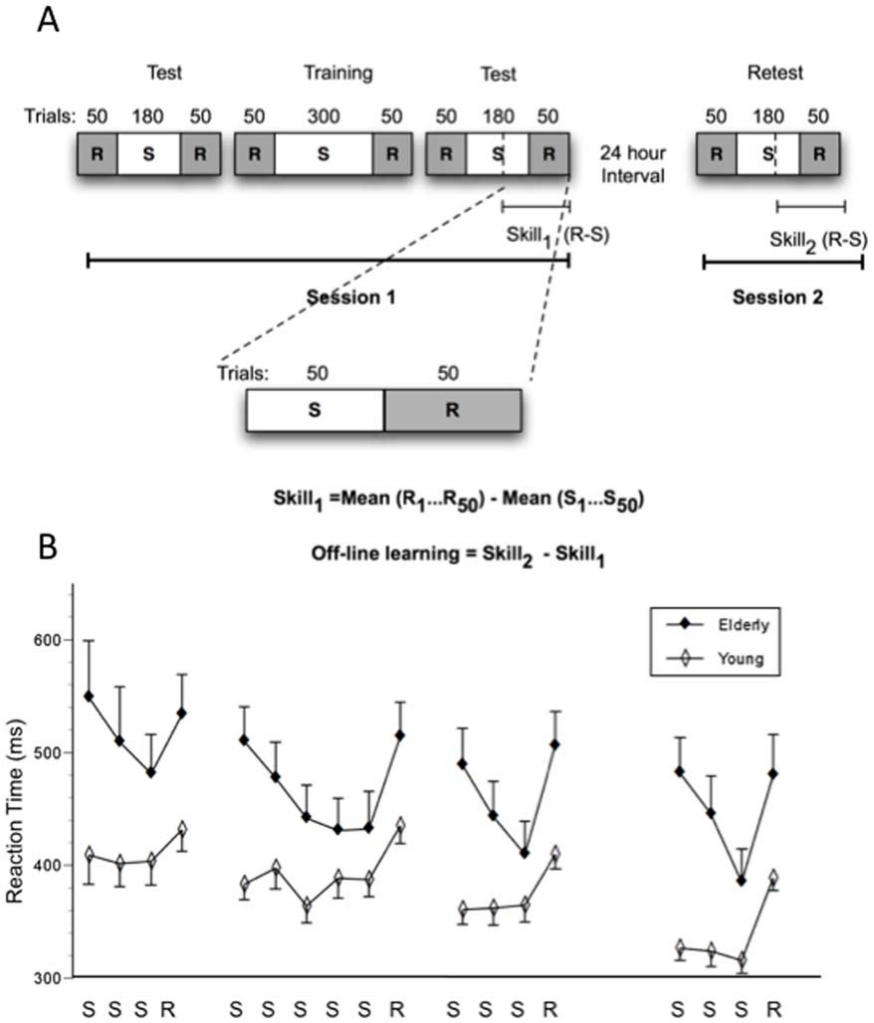
Study design and mean reaction times. A. Mean reaction times of 60 and 50 trials. The figure shows average reaction times of groups of 60 sequential trials and the last 50 random trials during each of the four testing blocks for younger and older subjects. Means of 60 are labeled “S” and means of 50 are labeled “R”. B. Serial Reaction Time Task (SRTT) design. The task was performed over four blocks, here labeled as test, training, test, and retest. The first three blocks of the task are completed during session 1, and the fourth block is completed during session 2. Each block begins and ends with 50 random trials (grey areas labeled “R”) sandwiching 180 or 300 sequence trials (white areas labeled “S”). A subject's skill at any given block is measured by subtracting the mean of the last 50 sequence trials from the mean of the last 50 random trials. Skill at the end of session 1, or block 3, is shown. The change in skill from session 1 to session 2 (“delta skill” or “off-line learning”) is found by subtracting skill at session 1 (Skill 1) from skill at session 2 (Skill 2). Error bars represent standard error of the mean.

The task began with 50 random trials, after which the 12-item sequence was introduced. This sequence repeated a set number of times before the dots would return to a random order. Participants were not informed of the existence of the sequence. Participants performed this task over three blocks during session 1, with a brief 1–2 minute rest between blocks, and a final block at session 2, 24 hours later. As shown in Figure 1B, each block began and ended with 50 random trials, with a series of sequence trials in the middle. The initial block contained 180 sequence trials (15 repetitions), the middle block contained 300 sequence trials (25 repetitions), and the final block of session one contained 180 sequence trials (15 repetitions). The fourth testing block completed at session 2, 24 hours later, contained 180 sequence trials.

After participants finished the fourth and final test block of the SRTT at the second testing session, they were immediately asked 1) if they noticed the sequence and 2) if they could recall the sequence. In previous studies using the SRTT, off-line skill improvements were affected by participants' free recall of the sequence. Those who recalled more than 8 items only showed off-line improvements over sleep, whereas those recalled 4-items or less demonstrated off-line improvements over both wake and sleep [9]. To remove this possible impediment to off-line skill improvements, participants who recalled more than 4 items of the sequence were excluded from analysis (*n* = 2).

After completing the entire SRTT task, participants also completed a test of declarative memory, the California Verbal Learning Test (CVLT-16), to contrast with our primary measure of procedural learning. This test requires participants to learn a list of 16 words over five oral presentations of the list. Participants are tested on 1) their free recall of the list immediately after each of the five oral presentations, 2) their free recall of the list after a short and a long delay (about 5 minutes and 20 minutes, respectively), and 3) their recognition of the words from a list of target and foil words.

## Results

### Skill Acquisition and Off-Line Improvement

Skill on the SRT task was defined as sequence-specific improvements demonstrated by declines in response time on sequence trials compared to the random trials which immediately followed. Only reaction times for correct responses were included for analysis of skill. To measure skill on the SRTT, the mean reaction times of the last 50 sequential trials and the 50 random trials that followed were contrasted at each of the four testing blocks (A, B, C and D) of the task [9], [12]. The effect of outlier trials were reduced by removing all reaction times that were more than three standard deviations away from the mean for each block. These outlying response times were replaced with the given testing block's mean reaction time [9]. This yielded a skill score for each block of the SRTT. To determine how much “off-line” learning (or “delta skill”) participants displayed, the skill at the end of session 1 (the skill at the third testing block) was subtracted from the skill at session 2 (skill at the fourth testing block).

**Figure 2:**
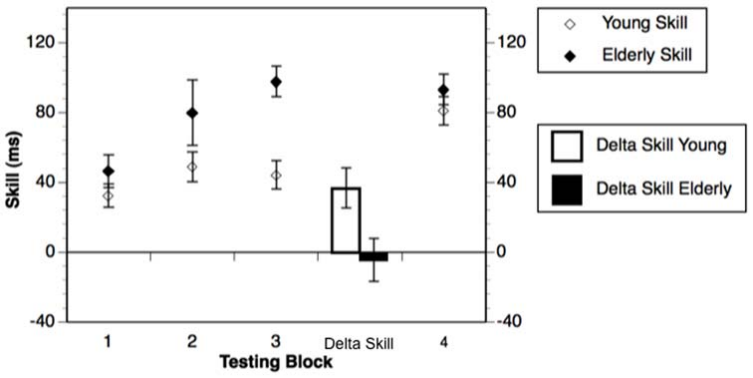
Skill by testing block and delta skill. The figure shows average skill at each of the four testing blocks as well as the change in skill from blocks 3 to 4 (or between test and re-test) for young and older participants. Error bars represent standard error of the mean. *Note*: **p<0.001; * *p*<0.05.

To examine any differences between young and older participants at session 1 and at re-testing, a two-way (Age Group: Young vs. Olders) X (Testing Session: Session 1 vs. Session 2) mixed Factors ANOVA was performed with age group as the between subjects factor, session as the within subjects factor, and skill as the dependent variable. Older participants showed higher average skill than the young (Main effect Age Group: *F*_(1, 24)_ = 4.92, *p*<0.05, *Older Mean* = 95.4±10.8; *Young Mean* = 62.6 ±10; all means will be reported±*SE*). As shown in Figure 2, at session 1 older participants showed significantly more skill than young participants (Interaction: *F*_(1, 24)_ = 6.00, *p*<0.05, Post hoc: *Older Mean* = 97.6±15.8, *Young Mean* = 44.2±6.9, *F*_(1, 24)_ = 20.05, *p*<0.001). At session 2, older participants' skill did not differ from that of the young (Post hoc: *Older Mean* = 93.1±15.6, *Young Mean* = 81±9.3, *F*_(1, 24)_ = 1.03, *ns*). In addition, based on our a priori hypotheses, we examined the change in skill between sessions for both young and older participants. Young participants showed an increase in skill from session 1 to session 2 (*Mean Delta Skill Young* = 36.8±11.4, *t*_(13)_ = −3.23, *p*<0.01), whereas older participants' skill did not change from session 1 to session 2 (*Mean Delta Skill Older* = −4.5±12.5, *t*_(11)_ = 0.37, *ns*). Young participants' change in skill was significantly greater than that of the older participants (*t*_(24)_ = −2.45, *p*<0.05 (see Figure 2)).

### Skill Acquisition and Off-Line Improvement as Percentage

As expected, older participants had slower reaction times (*M* = 464.2±30.1) than young participants (*M* = 393.9±13.8) irrespective of sequence and random trials (*t*_(24)_ = 2.23, *p*<0.05). To account for the possibility that older participants showed a greater difference in reaction times between sequence and random trials due to slower baseline reaction times, the percentage skill improvement was calculated across all four blocks of the task. Each participants' skill scores for each testing block of the SRTT was divided by their average random reaction time for that block, and the result was multiplied by 100 to obtain the percentage by which their reaction times decreased during the sequence trials. A two-way (Age group: Young vs Older participants) X (Testing session: Session 1 vs. Session 2) mixed-factors ANOVA was run using these scores, and similar results were found. Similar to the previous analysis, at session 1 older participants showed higher percent skill than the young participants (Interaction: *F*_(1, 24)_ = 6.32, *p*<0.05, Post hoc: *Older Mean* = 19%±2.4, *Young Mean* = 10.8%±1.6, *F*_(1, 24)_ = 10.19, *p*<0.01). Older and young participants' percent skill did not differ at session 2 (Post hoc: *Older Mean* = 19.5%±2.7, *Young Mean* = 20.5%±2.3, *F*_(1, 24)_ = 0.13, *ns*). Young participants also improved their percent skill from session 1 to session 2 (*Mean Delta Skill* = −9.6%±2.7, *t*_(13)_ = −3.51%, *p*<0.01) whereas the older participants showed no change in percent skill from session 1 to session 2 (*Mean Delta Skill* = −0.5%±2.3, *t*_(11)_ = −0.23, *ns*).

### Accuracy

To assess for the possibility of a speed-accuracy trade off, error-rate was examined over random and sequential trials of the SRT task for young and older participants. Error rate was calculated as a percentage of incorrect responses made by each participant out of the total number of responses they made during either random and sequencetial trials. For both age groups, error rates were greater during random trials than during sequential trials (*Main Effect Trial Type: F*_(1)_ = 22.29, *p*<0.05; *Mean Random* = 6.05±0.34, *Mean Sequential* = 4.15±0.34). Error rates did not differ significantly by age group (*Older Mean* = 4.42±0.92, *Young Mean* = 6.27±0.85, *F*_(1)_ = 2.18, *p* = ns), nor was there an interacting effect of age group and trial type on error rate (*F*_(1)_ = 0.01, *p* = ns). Neither age group appears to have sacrificed speed for accuracy or vice versa.

### Declarative Memory

Performance on the declarative memory task (CVLT) showed contrasting results to the implicit, procedural skill measure. Young participants, in contrast to their reduced skill measures, were better able to encode the list of words than older participants. Young participants correctly recalled more words over the five presentations of the 16-item list (*M* = 62.1±1.5, maximum score = 80) than the older participants (*M* = 51.4±3.4, *t*_(21)_ = −2.78, *p*<0.05). Young participants also correctly recalled more words after a 20-minute delay (*M* = 14.2±0.5) than the older participants (*M* = 11.4±1.1, *t*_(21)_ = −2.18, *p*<0.05). Young participants also correctly recognized more words from a list of foils (*M* = 15.1±0.4) than older participants (*M* = 10.7±1.5, *t*_(21)_ = −2.77, *p*<0.05).

## Discussion

Over a single practice session, older subjects acquired more skill on a sequence of finger movements than young subjects. This age discrepancy in skill is not attributable to the fact that older subjects are slower overall and thus have more opportunity to decrease their response times during the sequence trials, as expressing the skill as a percentage of baseline performance demonstrated the same results. The results also cannot be attributed to having selected older subjects with exceptional memory, as their scores on the declarative memory tasks were lower than those of the young.

As predicted, college-age subjects showed skill improvement over the 24-hour off-line period. The older participants showed no between-session improvement, but maintained their level of skill after 24 hours, which supports previous findings showing older adults' consistency of performance on motor tasks over long periods of time [4]. A ceiling effect could account for older adults' lack of off-line improvement, since older adults' initial skill was higher even than young adults' skill at re-test. Further investigation is needed to determine whether older adults can demonstrate enhancement of motor skills off-line.

The finding that older participants gained more skill than young participants at session one was unexpected, as previous studies have reported that older participants show magnitudes of sequence-specific learning that are, at most, equal to that of young participants over initial practice[5], [6]. This discrepancy of findings could be due to the current older sample being younger (55–70 years) than previous older samples (approx. 60–79 years, [11]; approx. 65–80 years, [5], [6], [13]. However, our sample may have been appropriate for examining normal aging separately from extraneous cognitive declines. Strict screening was applied to exclude subjects with either dementia or mild cognitive impairment, and older subjects were also matched closely to young in terms of education. Furthermore, despite the younger age range, our older sample showed characteristically poorer declarative memory than the young adults as well as slower reaction times. The current sample of older adults may therefore be representative of normal aging in the absence of significant pathology.

The current demonstration of older adults' superior skill could be suggestive of possible interacting memory systems, particularly between the systems that support declarative memory and those that support procedural memory. Some studies have presented evidence for interacting memory systems by showing that disruption of one system can lead to enhancement in the other, and vice versa [14], [15]. Such an interaction might predict that declines in declarative memory, such as those that occur with age, would lead to enhanced procedural memory. Even normal aging is associated with hippocampal atrophy and decreased activation in imaging studies[16]. Conversely, motor regions including primary motor cortex, premotor cortex, cerebellum and the supplementary motor area show compensatory increases in activation with normal aging [17]. Either declarative memory impairment or increased activation in motor networks could underlay our findings.

In summary, we found that older adults can actually acquire greater sequence skill during practice than college-age students. This difference could not be ascribed to older adults' slower overall reaction times or to selection of older adults with exceptional memory. As previously shown, the young showed off-line improvements between sessions, but these only brought the young up to comparable skill levels to the older adults. At least under certain circumstances, older adults can actually show greater acquisition of skill than young. The effect of aging on skill consolidation is unclear, yet the fact that participants maintained their skill levels after 24 hours suggests that their skill may stabilize over the off-line period even if it may not be enhanced as it is for the young.
